# Impaired Recognition of Basic Emotions from Facial Expressions in Young People with Autism Spectrum Disorder: Assessing the Importance of Expression Intensity

**DOI:** 10.1007/s10803-017-3091-7

**Published:** 2017-03-31

**Authors:** Sarah Griffiths, Christopher Jarrold, Ian S. Penton-Voak, Andy T. Woods, Andy L. Skinner, Marcus R. Munafò

**Affiliations:** 1 School of Experimental Psychology, University of Bristol, Bristol, UK; 2 MRC Integrative Epidemiology Unit (IEU), University of Bristol, Bristol, UK; 3 Xperiment, Surrey, UK; 4 UK Centre for Tobacco and Alcohol Studies, University of Bristol, Bristol, UK; 5 Autism Research Centre, Department of Psychiatry, University of Cambridge, Cambridge, UK

**Keywords:** Emotion recognition, Intensity, Perception, Bias, Facial expression, Online testing

## Abstract

It has been proposed that impairments in emotion recognition in ASD are greater for more subtle expressions of emotion. We measured recognition of 6 basic facial expressions at 8 intensity levels in young people (6–16 years) with ASD (N = 63) and controls (N = 64) via an Internet platform. Participants with ASD were less accurate than controls at labelling expressions across intensity levels, although differences at very low levels were not detected due to floor effects. Recognition accuracy did not correlate with parent-reported social functioning in either group. These findings provide further evidence for an impairment in recognition of basic emotion in ASD and do not support the idea that this impairment is limited solely to low intensity expressions.

**Electronic supplementary material:**

The online version of this article (doi:10.1007/s10803-017-3091-7) contains supplementary material, which is available to authorized users.

A core feature of autism spectrum disorder (ASD) is impairments in social communication and social interaction (American Psychiatric Association 2013). Difficulty identifying emotions from other people’s facial expressions may contribute to these impairments (Hobson 1995). This idea has been supported by studies finding associations between the ability to identify facial expressions and social skills in individuals with ASD (Wallace et al. 2011; Williams and Gray 2012) and studies showing that interventions targeting facial expression recognition lead to improvements in social skill in children with ASD (Rice et al. 2015; Thomeer et al. 2015). However, research aiming to demonstrate an impairment in facial expression recognition in individuals with ASD has provided mixed results.

While many studies have found evidence for poorer recognition of emotions from facial expression in ASD (Boraston et al. 2007; Evers et al. 2015; Humphreys et al. 2007; Lindner and Rosen 2006; Rump et al. 2009), others have found no evidence for this difference (Grossman and Tager-Flusberg 2012; Jones et al. 2011; Loveland et al. 1997; Tracy et al. 2011). These mixed findings may be due to differences in the tasks used in different studies (Harms et al. 2010). Some studies that have found no impairment have only tested recognition of emotion from high intensity (more exaggerated) expressions (Jones et al. 2011; Tracy et al. 2011). Individuals with ASD may use compensatory cognitive or linguistic strategies, rather than automatic affective processing, to try to recognise expressions (Grossman et al. 2000; Rutherford and McIntosh 2007). These compensatory strategies may only be effective when task demands are low, allowing for accurate emotion recognition, but ineffective when tasks demands are higher, for example when the emotional expressions are more subtle (Rump et al. 2009; Wallace et al. 2011; Wong et al. 2012) or presentation times are shorter (Clark et al. 2008).

A number of studies have directly compared recognition of different intensity expressions, testing the idea that impairments in recognition are only evident for subtle expressions. Wong et al. (2012) found that children with ASD were impaired at recognising photos of expressions rated as low intensity relative to children without ASD, but not at recognising photos of expressions rated as high intensity. Other studies have tested recognition of a larger range of expression intensities by morphing neutral expressions with full intensity expressions to create sequences of static expressions varying in intensity. Using static images from these sequences, Doi et al. (2013) found impairment in adults with ASD in labelling happiness and sadness at medium intensity levels but not high or low intensity levels. A number of studies have used intensity sequences to show neutral expressions dynamically morphing to reach different intensity levels. Using such dynamic sequences, Law Smith et al. (2010) found impaired recognition of low intensity, but not high intensity expressions of anger and surprise in adults with ASD. However, other studies using similar dynamic morph sequence stimuli have found no impact of expression intensity on impairment in emotion recognition from facial expressions (Evers et al. 2015; Kessels et al. 2010; Ketelaars et al. 2016).

A failure to account for response biases in forced choice expression labelling tasks may lead to inconsistent results between studies (Evers et al. 2015). Response biases towards negative emotions are common in mood disorders such as depression and anxiety (Bell et al. 2011; Bourke et al. 2010), which often co-occur with ASD (Joshi et al. 2010). Response biases towards negative emotions have also been found in ASD in forced choice labelling tasks (Eack et al. 2015; Evers et al. 2015). In real life, expressions are often low intensity and ambiguous, so response biases may have a relatively large effect on emotion processing in everyday interactions. Testing recognition using low intensity expressions allows accurate measurement of biases, which can then be accounted for when assessing differences in accuracy between groups.

A further methodological reason for the mixed evidence for an impairment in emotion recognition in ASD, is that many studies in this field lack statistical power. A meta-analysis of 48 studies of emotion recognition concluded that a global impairment did exist (Uljarevic and Hamilton 2012), but that the effect size for a group difference after correction for publication bias was modest (*d* = 0.4). Previous studies that have found that the impairment depends on the intensity level of the expressions have had very small sample sizes (often <20 in the ASD group). Further research with larger sample sizes is needed before it can be concluded that impairments in emotion recognition in ASD are larger for lower intensity expressions.

The current study aimed to compare a group of young people with ASD and a typically developing control group on recognition of facial expressions from morph sequences that vary the intensity of expressed emotion. An online testing platform was used in order to collect data from a large sample, to ensure sufficient statistical power to detect a modest group difference. We hypothesised that there would be an impairment in recognition of the 6 basic facial expressions in the group with ASD, in comparison to the control group (Uljarevic and Hamilton 2012), that was greater at lower intensity levels than higher intensity levels (Doi et al. 2013; Law Smith et al. 2010; Wong et al. 2012). Additionally we hypothesised that emotion recognition ability would be correlated with parent-reported social skills in the ASD group (Wallace et al. 2011; Williams and Gray 2012). The protocol for this study was preregistered on the Open Science framework (https://osf.io/i6dhf/).

## Method

### Participants

Participants with ASD were recruited via the Autism Spectrum Database UK (http://www.ASD-UK.com), adverts placed on autism charity websites, emails to parent support group mailing lists, visits to parent support group meetings, and word of mouth. Control participants were recruited via the University of Bristol Cognitive Development Centre participant database and word of mouth. All participants had to be between 6 and 16 years old, be native English speakers and have normal or corrected to normal vision. Participants in the ASD group had to have a diagnosis given by a professional clinician. Participants in the control group had to have no diagnosis of ASD or any learning disability, and no first-degree relatives with ASD. Parents of all children provided informed consent on the study website prior to their child starting the study tasks. All participants had their names entered into a prize draw to win a tablet computer. The study was approved by the University of Bristol Faculty of Science Human Research Ethics Committee.

### Measures

#### Emotion Recognition Task (ERT)

**Fig. 1:**
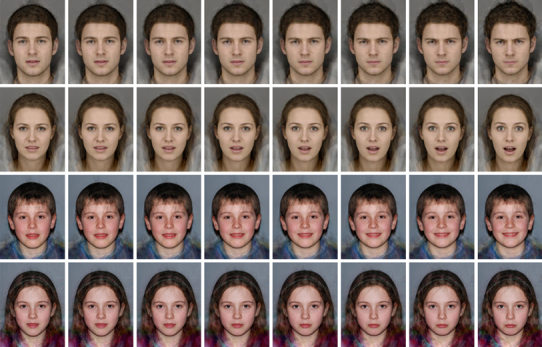
Examples of morph sequence stimuli from low intensity (*left*) to high intensity (*right*). From *top* to *bottom*; male adult angry sequence, female adult surprise sequence, male child happy sequence, female child sad sequence

The expression recognition task was a forced choice labelling task that included faces displaying the 6 basic emotional expressions (happy, sad, angry, disgusted, scared and surprised; Ekman 1992) at 8 different intensity levels. Stimuli were prototype faces, created by averaging photos of 12–15 individuals of the same age and gender posing the same facial expression using the program Psychomorph (Tiddeman et al. 2001). Four different face prototypes were produced for each emotion expression: male adult, female adult, female child and male child (see Fig. 1). Griffiths et al. (2015) provides a detailed description of the creation of the prototypes including collection of original photos. For each face prototype an emotionally ambiguous expression was created by averaging all expression images (Skinner and Benton 2010). These emotionally ambiguous expressions were used to create 8-step morph sequences running between the ambiguous expression and each full intensity emotional expression for each face prototype, resulting in 192 stimuli. We chose to create sequences from an ambiguous expression rather than a neutral expression to ensure that the starting point of the sequences was equidistant from all of the emotions in perceptual space. Neutral expressions do not lie at the centre of perceptual face space (Shah and Lewis 2003) and are perceived as negative rather than emotionally neutral (Lee et al. 2008). Each of the 192 stimuli were presented once in the task in a random sequence. Stimuli were presented in 4 blocks of 48 trials.

On each trial participants were presented with a fixation cross on the screen for a random interval between 1500 and 2500 ms, followed by the stimulus for 300 ms. This short presentation time was chosen to minimise use of compensatory strategies and was shown to be acceptable in piloting with children with ASD. A visual mask was then presented for 150 ms, after which the 6 emotion labels appeared in a circular formation on the screen. Participants used the curser to respond by selecting the emotional label that they thought best described the emotion of the face. The labels remained on the screen until the participant made a response. The position of the emotion labels was randomly selected for each participant but remained consistent for that participant throughout the testing session.

#### Receptive One-Word Picture Vocabulary Scale

The Receptive One-Word Picture Vocabulary Test (ROWPVT; Martin and Brownell 2011) is a vocabulary test designed for individuals aged from 2 up to 80+ years. The test was adapted for online administration with permission from the publishers. On each trial participants heard a recording of a single spoken word and were shown four pictures on the screen. Participants used the mouse to select the picture that matched the spoken word. Score on the ROWPVT was used as a covariate in data analyses to remove any variance in expression labelling performance that could be attributed to verbal ability.

#### Ravens Progressive Matrices

Raven’s Standard Progressive Matrices (RPM; Raven et al. 2000) is a 60-item measure of non-verbal reasoning designed for a range of ages and abilities. It was adapted for online administration with permission from the publishers. On each trial, participants were presented with a geometric pattern with a piece missing and a choice of 4–6 shapes to complete the pattern. Participants used the mouse to select the correct shape to complete the pattern. Performance on the RPM was used as a covariate in data analyses to remove any variance in expression labelling performance that could be attributed to non-verbal cognitive ability.

#### Social Communication Questionnaire (lifetime)

The lifetime version of the Social Communication Questionnaire (SCQ; Rutter et al. 2003) is 40-item parent-report questionnaire that asks about a child’s developmental history, in order to measure ASD diagnostic traits. Scores indicate the likelihood that a child has an ASD. The SCQ has been shown to reliably discriminate children with ASD from those without ASD within the general population (Chandler et al. 2007). Score on the SCQ was used in this study to assess the level of autistic traits in each group and also to determine whether autistic traits correlated with performance on the emotion recognition task.

### Procedure

Parents who expressed an interest in their child taking part in the study were given a unique password for the study website Xperiment (http://www.xperiment.mobi). The study website was compatible with desktop or laptop computers but not tablets or phones. Once logged in, parents were provided with full information about the study and asked to complete the parental consent form. After consent was collected, children could start the study tasks. The order of the tasks was constrained so that participants first completed the emotion recognition task followed by the ROWPVT and finally the RPM. Participants could not move on to the next task without completing the previous task. Parents were instructed not to look at the screen whilst their child was completing the tasks in order to avoid them influencing their child’s responses. Participants were able to log off in between each task, and to log back in to complete the remaining tasks on separate days. Parents were also asked to complete an online questionnaire on a different website which included questions about demographics, questions to confirm their children’s eligibility for the study, and the items of the SCQ. The questionnaire could be completed at any point during the study. Parents were sent periodic emails to remind them to log in to each site and complete any remaining tasks.

### Statistical Analysis

The dependent variable in our analysis of emotion recognition accuracy was unbiased hit rate (Hu; Wagner 1993). Raw hit rate is affected by any imbalance in participants’ tendency to select a particular emotional response. For example, if a participant has a bias towards selecting the “happy” label, independent of the emotion presented, their hit rate for recognition of happiness will be inflated. It is therefore important to control for response bias when calculating accuracy in category judgement paradigms. The unbiased hit rate transformation was devised to account for response bias in category judgement paradigms when other methods of adjusting for response bias (i.e. signal detection) are unsuitable due to the nature of the categories. Hu is calculated as: Hu = (Ai/Bi) × (Ai/ Ci), where Ai = number of hits, Bi = number of trials where i is target and Ci = frequency of i responses (hits and false alarms). Analyses were also conducted with raw hit rate as the dependent variable, the results of which can be found in the supplementary material.

Our primary analysis compared groups on their accuracy for recognising each emotion at each intensity level. As specified in the preregistered study protocol, this was achieved by entering accuracy into a 2 × 6 × 8 mixed model ANOVA, with group (ASD, control) as a between-subjects factor, and emotion (happy, sad, angry, disgusted, fearful, surprised) and intensity level (1–8) as within-subjects factors. In order to understand any interactions that were supported by the 3-way ANOVA, we conducted 2-way ANOVAs on accuracy for each emotion separately, with group and intensity level as factors. Analyses were conducted unadjusted and adjusted for ROWPVT, RPM and participant gender. Where the results do not differ qualitatively, statistical values are reported for the adjusted analyses only (see supplementary material for values from the unadjusted analyses).

The secondary analysis specified in the study protocol was a correlation analysis to determine whether participants’ social functioning, as indexed by their score on the SCQ, was associated with overall accuracy on the emotion recognition task. Additionally, given some evidence that children with ASD may be more impaired at recognising emotion on adults faces (Lerner et al. 2013), we tested whether the age of the face stimuli differentially affected the performance of the two groups by running a 2 × 6 × 2 mixed model ANCOVA with group as a between-subjects factor and emotion and face age (child, adult) as within-subjects factors. Analyses were conducted unadjusted and adjusted for ROWPVT, RPM and participant gender (see supplementary material for values from the unadjusted analyses).

Sample size was based on a power calculation using the effect size for a global emotion recognition impairment in ASD reported in the meta-analysis by Uljarevic and Hamilton (2012), which was estimated to be *d* = 0.4 after adjusting for publication bias in the literature. This indicated that 78 participants would be required in each group to achieve 80% power, and 108 in each group to achieve 90% power, to detect this effect at an alpha level of 0.05.

The data that form the basis of the results presented here are archived on the data.bris Research Data Repository DOI: 10.5523/bris.7cl78lm43ate1b0qo7yprz6p4.

## Results

### Participants

**Fig. 2:**
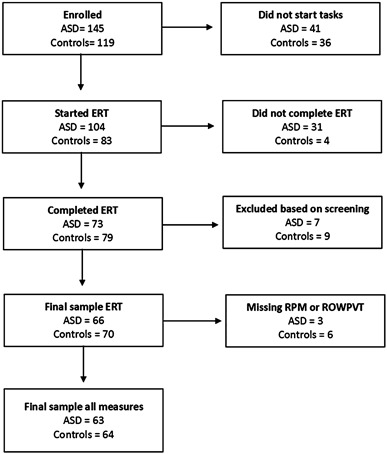
CONSORT diagram showing numbers of participants recruited and numbers of participants that completed each stage of the study

We surpassed our recruitment objective, recruiting a total of 145 participants with autism and 119 controls. However, a substantial number of participants dropped out after being enrolled (see Fig. 2.). Notably, it was much more common for participants with ASD to drop out after attempting the ERT. A small number of participants that completed the ERT were excluded from the analysis. This included five participants with ASD and six without ASD who were excluded because their parents did not complete the demographics questionnaire, meaning their eligibility could not be confirmed. A further two participants with ASD and three without ASD were excluded due to ineligibility for the following reasons; visual impairment (N = 1 with ASD), lack of official ASD diagnosis (N = 1 with ASD), first degree relative with ASD (N = 1 without ASD), diagnosis of learning disability (N = 2 without ASD). RPM and ROWPVT data were missing for some participants due to drop-out and technical errors. These participants were therefore not included in the main analyses which adjusted for these variables, although they were included in the correlational analyses assessing the relationship between SCQ and overall performance. This left a total of 63 participants with ASD and 64 participants without ASD for the main analysis, and 66 participants with ASD and 70 participants without ASD for the correlational analysis. Although we did not achieve the intended sample size, the achieved sample size gave us 72% power to detect a difference between groups in our main analysis based on the estimated effect size from previous research (Uljarevic and Hamilton 2012). For those in the final sample, the average number of days between completion of the first and final task was 8 days (SD = 17 days) for the ASD group and 6 days (SD = 11 days) for the control group.

Table 1 shows summary statistics for each group for age, gender, raw scores on RPM, ROWPVT and SCQ. These data suggest that the groups were well matched on age but that the ASD group had lower verbal ability and non-verbal reasoning ability than the controls. Additionally, there was a much higher proportion of males in the group with ASD (88%), compared to the control group (50%), as would be expected given the higher prevalence rate of ASD in males than females. As expected, the ASD group scored much higher on the SCQ, with 55/66 scoring above the cut-off and 68/70 of the control participants scoring below the cut-off for likely ASD diagnosis. All analyses were rerun excluding participants with ASD who fell below the cut off and control participants who fell above the cut off. Analyses were also rerun including only male participants in both groups. Both of these additional analyses showed broadly similar results to the analysis of the full sample (see supplementary materials).

**Table 1:** Participant characteristics for the ASD group and typically developing control group

		ASD	Control	
Age	Mean (SD)	11.24 (2.91)	11.24 (2.49)	*p* = .999
	n (males)	66 (58)	70 (35)	
SCQ	Mean (SD)	21.50 (7.13)	3.63 (4.13)	*p* < .001
	n (males)	66 (58)	70 (35)	
RPM	Mean (SD)	37.08 (11.61)	40.16 (9.72)	*p* = .108
	n (males)	63 (55)	64 (30)	
ROWPVT	Mean (SD)	127.14 (24.26)	135.17 (24.99)	*p* = .069
	n (males)	63 (55)	64 (30)	

*Note* All scores are raw scores. *p*-values are given for independent sample *t* tests

All participants in the final ASD group had received a diagnosis of an ASD from a paediatrician (58%), clinical psychologist (18%), educational psychologist (12%) or other qualified professional. The majority had received their diagnosis in a UK National Health Service (88%), with a small number receiving a diagnosis at school (6%) or from a private clinic (6%). The majority of participants were in mainstream education (76%) or in a specialist education unit within a mainstream school (14%), with a small number attending specialist school (4%), or being home educated (6%).

### Primary Analysis of ERT Performance

Unadjusted analyses of unbiased hit rate showed clear evidence for a main effect of group [*F*(1, 125) = 15.61, *p* < .001, *ɳ*^*2*^ = 0.030], with the ASD group being less accurate than the control group. However, there was little evidence for this overall group difference once the analysis was adjusted for ROWPVT, RPM and participant gender [*F*(1, 125) = 1.48, *p* = .23, *ɳ*^*2*^ = 0.003]. In both adjusted and unadjusted analyses there was strong evidence for main effects of emotion [adjusted; *F*(5, 625) = 141.47, *p* < .001, *ɳ*^*2*^ = 0.146] and intensity [adjusted; *F*(7, 875) = 472.54, *p* < .001, *ɳ*^*2*^ = 0.435], and for interactions between group and emotion [adjusted; *F*(5,625) = 4.51, *p* < .001, *ɳ*^*2*^ = 0.005], group and intensity [adjusted; *F*(7, 875) = 5.30, *p* < .001, *ɳ*^*2*^ = 0.009], emotion and intensity [adjusted; *F*(35, 4375) = 14.46, *p* < .001, *ɳ*^*2*^ = 0.046], with some evidence of an interaction between group, emotion and intensity [adjusted; *F*(35, 4375) = 1.53, *p* = .023, *ɳ*^*2*^ = 0.005].

Follow up analyses for separate emotions provided some evidence for a main effect of group for all emotions in the unadjusted analysis [*F*s(1,125) > 3.13, *ps* < 0.079, *ɳ*^*2*^ > 0.010] but only for disgust in the adjusted analysis [*F*(1, 125) = 7.68, *p* = .006, *ɳ*^*2*^ = 0.025, all others; *F*s(1, 125) < 1.04, *ps* > 0.31, *ɳ*^*2*^ < 0.004]. In both analyses there was strong evidence of a main effect of intensity for all emotions [adjusted; *F*s(7, 875) > 54.59, *p*s < 0.001, *ɳ*^*2*^ > 0.213]. Crucially, in both adjusted and unadjusted analyses, there was evidence for interactions between group and intensity for anger [adjusted; *F*(7, 875) = 2.58, *p* = .012, *ɳ*^*2*^= 0.013], disgust [adjusted; *F*(7, 875) = 5.90, *p* < .001, *ɳ*^*2*^ = 0.027], sadness [adjusted; *F*(7, 875) = 3.24, *p* = .002, *ɳ*^*2*^ = 0.017] and surprise [adjusted; *F*(7, 875) = 2.06, *p* = .046, *ɳ*^*2*^ = 0.010], weak evidence for happiness [adjusted; *F*(7, 875) = 1.72, *p* = .10, *ɳ*^*2*^ = 0.008], but no clear evidence for fear [adjusted; *F*(7, 875) = 1.07, *p* = .38, *ɳ*^*2*^ = 0.005].

**Fig. 3:**
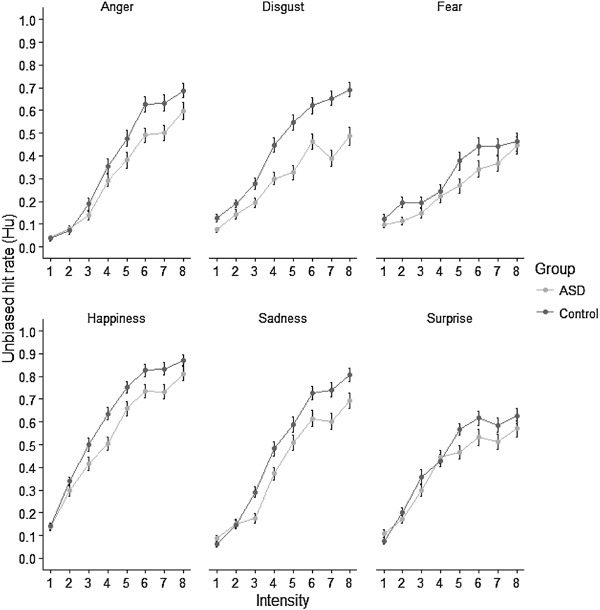
Unbiased hit rate for each emotion at each intensity level for each group. *Error bars* represent standard error

From inspection of the data in Fig. 3, it is clear that recognition of the lowest intensity expressions of all emotions was very poor in both groups. Interactions between group and intensity in our main analysis may therefore have been driven by floor effects that preclude the detection of group differences in accuracy at the lowest intensity levels. We therefore carried out a follow up analysis, in which we reran the adjusted ANOVA only including the highest 6 intensity levels, to test whether the interactions between group and intensity were being driven by floor effects at the lowest two intensity levels.

The reanalysis did not provide strong evidence for an interaction between group and intensity [*F*(1, 625) = 1.57, *p* = .166, *ɳ*^*2*^ = 0.002] or group, emotion and intensity [*F*(25, 3125) = 1.24, *p* = .187, *ɳ*^*2*^ = 0.004], suggesting that interactions between group and intensity in the main analyses were likely being driven by floor effects at very low intensity levels. There was still no clear evidence for a main effect of group [*F*(1, 125) = 1.44, *p* = .232, *ɳ*^*2*^ = 0.003], but there was clear evidence of a two-way interaction between group and emotion [*F*(5, 625) = 4.54, *p* < .001, *ɳ*^*2*^ = 0.007]. As in the full analysis, there was evidence for a main effect of emotion [*F*(5, 625) 137.04, *p* < .001, *ɳ*^*2*^ = 0.172), and intensity [*F* (*5*,625) = 232.50, *p* < .001, *ɳ*^*2*^ = 0.225] and for an interaction between emotion and intensity [*F*(25,3125) = 6.17, *p* < .001, *ɳ*^*2*^ = 0.018].

In order to determine what was driving the group by emotion interaction in this reanalysis, we collapsed the data across the 6 intensity levels and compared group performance for each emotion in between-subjects t-tests. These provided good evidence for a group difference for anger [*t*(125) = 2.10, *p* = .003], disgust [*t*(125) = 5.56, *p* < .001], happiness [*t*(125) = 3.27, *p* = .001] and sadness [*t*(125) = 3.63, *p* < .001], and weak evidence for a group difference for surprise [*t*(125) = 1.92, *p* = .057] and fear [*t*(125) = 1.86, *p* = .065]. Taken together these results suggest that participants with ASD are less accurate than controls at recognising emotions from expressions at various intensity levels, once the expressions are intense enough to allow performance beyond floor levels.

**Fig. 4:**
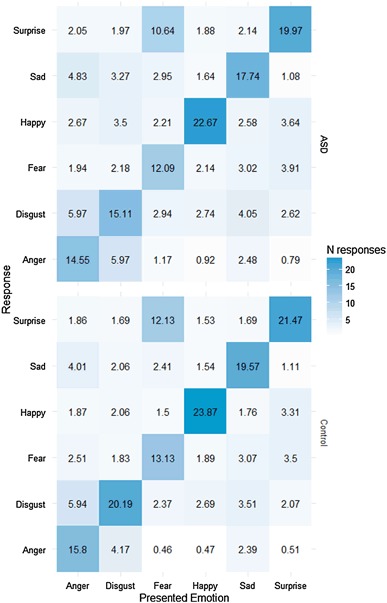
Confusion matrices of mean number of responses in each emotion category for stimuli showing each emotion, for the ASD group (*top*) and control group (*bottom*)

To explore whether there were any systematic group differences in error patterns, we calculated a confusion matrix for responses for each emotion (collapsed across all 8 intensity levels) for each group (see Fig. 4). Visual inspection of the data suggests that the ASD and control group show very similar confusion patterns. This was confirmed using a Chi-squared test comparing the two groups’ average number of misattributions of each emotion label, which provided no evidence for a difference between groups [*χ*^*2*^(5) = 0.69, *p* = .98].

### Correlational Analysis

There was no clear evidence for the predicted negative correlation between overall accuracy on the emotion labelling task and score on the SCQ in either the ASD group (*r*_*s*_ = −0.043, *p* = .73) or the control group (*r*_*s*_ = −0.092, *p* = .45), suggesting no strong relationship between emotion recognition ability and parentally-assessed level of social functioning using the SCQ in either group.

### Face Age Manipulation Check

We found no evidence for critical interactions between face age and diagnostic group (*ps* > 0.66) suggesting that face age did not differentially influence the performance of the two groups for recognition of any emotion.

## Discussion

We found that children and adolescents with ASD were less accurate than controls at recognising emotion from intense as well as more subtle expressions. However, we did not find evidence for a group difference in recognition of expressions at very low intensity levels due to floor effects. The difference in performance between groups was evident for all basic emotions although the statistical evidence for a group difference was weaker for recognition of fear and possibly surprise. For fear, accuracy was low in both groups, even at the higher intensity levels, suggesting the lack of group difference here may be due to poor performance in both groups. The current results therefore corroborates the existence of a small but broad impairment in recognition of basic emotions in ASD (Uljarevic and Hamilton 2012), which is present across different intensity expressions, once performance rises above floor levels.

We had predicted that we would find a smaller impairment in the ASD group for recognition of high intensity expressions compared to more subtle, low or intermediate intensity expressions. Instead, we found that intensity did not have a large influence on the group differences, once performance on very low intensity expressions, where recognition was very poor in both groups, had been excluded. Finding a lack of group difference at very low levels is unsurprising given these expressions are highly ambiguous. Previous studies that have used expressions that vary in intensity have found no group differences in recognition at very low intensity levels for the same reason (Doi et al. 2013). However, based on previous research, we expected that the group difference that appeared at intermediate levels might reduce as the expression increased in intensity (Doi et al. 2013; Law Smith et al. 2010; Wong et al. 2012). Our results are however, consistent with another recent study that found an impairment in recognition of basic emotions from dynamic expression sequences that was consistent across different intensity levels (Evers et al. 2015). Notably, that study and the current study both have sample sizes over twice as large as the previous studies that found a reduction in impairment in recognition of high intensity expressions. Based on these results we suggest that the issue of intensity may not be as important a factor in recognition impairment in ASD as has previously been suggested, and that previous evidence of a moderating effect of intensity reflects the combination of a lack of statistical power to consistently detect a subtle deficits and floor/ceiling effects on performance.

There are, however, a number of factors to consider before dismissing the issue of expression intensity influencing emotion recognition impairment in ASD completely. First, it is difficult to directly compare intensity levels across studies. Even full intensity expression (sometimes referred to as 100% intensity) has not been objectivity matched. It is therefore possible that the ‘high’ intensity expressions in this study may have been similar to the ‘medium’ intensity levels in previous studies. If we had extended the morph sequences to include more exaggerated expressions we may have eventually found a reduction in impairment. Second, good performance in groups with ASD at high intensity levels is attributed to cognitive or linguistic compensatory strategies that are effective only at high intensity levels and take longer to implement than automatic affective processing (Clark et al. 2008; Doi et al. 2013; Grossman et al. 2000; Rutherford and McIntosh 2007). The short presentation times used in this study (300 ms) may have prevented the use of such compensatory strategies, revealing impairments in recognition even for relatively high intensity expressions. Future studies could vary intensity and presentation time to determine if these two factors interact to predict performance of individuals with ASD.

We found that the group with ASD produced a very similar pattern of errors to the control group (see Fig. 4). Both groups frequently confused disgust and anger for each other, and mistook fear for surprise, a confusion pattern that is common in forced choice expression labelling tasks in typically developing individuals (Dalrymple et al. 2013). The similarity of group response patterns suggests that there is no systematic response bias at a group level that causes reduced accuracy in recognition in ASD. Two recent studies have suggested a bias towards particular negative emotions among adults (Eack et al. 2015) and children (Evers et al. 2015) with ASD. In contrast, our results align with a number of other studies that have found no evidence for differences in confusion patterns (Jones et al. 2011; Wallace et al. 2011; Wong et al. 2012), suggesting that those with ASD are generally using the same types of cue to identify emotion from expressions, even if they are slightly less accurate in doing this (Wallace et al. 2011).

Unexpectedly we did not find a meaningful correlation between overall performance on the emotion recognition task and social functioning, as measured by parent responses to the lifetime version of the Social Communication Questionnaire. This is somewhat surprising as many other studies have found evidence for a relationship between emotion recognition performance and social functioning using similar measures (Evers et al. 2015; Wallace et al. 2011; Williams and Gray 2012). However, we did not power this study to look for this correlation, and, given that the relationship was in the correct direction, it is possible that there is a small correlation between emotion recognition and social functioning that we did not find statistical evidence for due to a lack of statistical power.

A substantial limitation of this study is that we did not achieve the intended sample due to an unexpected degree of participant attrition during testing. Thirty percent of participants with ASD dropped out after starting the emotion recognition task, which is more than one might have expected had this been a laboratory-based task. It is not entirely clear why this was the case, but perhaps participants are more motivated to continue with a task that is repetitive or difficult if they are in a laboratory environment rather than at home. This is a potential concern as it may mean that those who found the task harder were less likely to continue with the task, reducing the size of the impairment that we found. Nonetheless, despite the drop out, we still achieved 72% power to detect a difference between groups. This study is still one of the larger studies to look at emotion recognition in young people with ASD, and the largest to look at the effect of expression intensity on the recognition impairment.

A further limitation is that, due to testing online, were not able to formally confirm diagnosis with gold standard diagnostic measures (e.g., autism diagnostic observation schedule; ADOS; Lord et al. 2000). However, participants’ parents reported that they had a diagnosis of ASD from a qualified professional. Given there was no great financial incentive to take part, we feel it is unlikely that parents would have misreported their child’s diagnosis. Furthermore, we reanalysed our results, removing those who did not score above the cut off for ASD on the SCQ (e.g., those who arguably were the least likely to have a reliable diagnosis) and this did not change our results.

The gender ratios in our groups were not equivalent as our group with ASD was predominantly (88%) male, while our control group had an equal gender ratio. This may have influenced our results, as females have been shown to perform better at emotion recognition tasks (Thompson and Voyer 2014). We included gender in our analysis to adjust for unequal gender ratios between groups, as is common in studies of emotion recognition where groups have unequal gender ratios (for example; Anderson et al. 2011). However, the evidence for a group difference in the adjusted analysis was weaker than in the adjusted analysis, suggesting that gender may have accounted for some of the difference in emotion recognition accuracy between the ASD and control group.

The current study is one of the first to carry out cognitive testing of this age group with a diagnosis of ASD over the Internet (see Sucksmith et al. 2013 for an example of online testing of emotion recongition in adults with ASD). Despite piloting the study procedure carefully and adding in measures to limit potential concerns (such as instructions to reduce parental influence), we cannot be certain of the reliability of Internet testing in this population. However, internet testing has been shown to be reliable for perception experiments in the typical population (Germine et al. 2012) and the fact that we found results that were broadly in line with the results of a meta-analysis of laboratory studies (Uljarevic and Hamilton 2012) gives us confidence in the validity of our Internet-based tasks.

Online testing has several advantages over testing in the laboratory. Participants can be recruited from across a large geographical area, increasing the pool of potential participants and therefore sample size (as long as drop out can be limited). Furthermore, and perhaps of particular relevance in research with individuals with ASD, participants can complete the study in comfortable and familiar surroundings, potentially increasing participation of a subset of the population who would be uncomfortable coming in to the lab. These advantages must be weighed up against the potential disadvantages of greater attrition in online tasks compared to lab based tasks, and the use of parent or self-reported ASD diagnosis, rather than gold standard diagnostic assessments.

In conclusion, this study provides further evidence that there is a small reliable impairment in recognition of basic facial expression in ASD that is evident when expressions are presented for limited durations. The impairment is consistent across different intensity levels, once expressions are intense enough to allow performance above floor levels. This is at odds with the idea that there is a particular deficit in recognition of low intensity expressions (Wong et al. 2012). Training schemes for improving emotion recognition in ASD should therefore include full intensity basic expressions, perhaps presented only briefly, as well as more subtle or complex emotions that might allow generalisation to the complexity of real social interactions.

## Electronic supplementary material

Below is the link to the electronic supplementary material.

Supplementary material 1 (DOCX 20 KB)
