# Supplementary material for: Impaired Recognition of Basic Emotions from Facial Expressions in Young People with Autism Spectrum Disorder: Assessing the Importance of Expression Intensity
Source: J Autism Dev Disord. 2017 Mar 31;49(7):2768–78. doi: 10.1007/s10803-017-3091-7 (PMC6606653; doi:10.1007/s10803-017-3091-7)
Supplement: Supplementary file 1 — Supplementary material 1 (DOCX 20 KB) [file 10803_2017_3091_MOESM1_ESM.docx]

*Unadjusted analyses of unbiased hit rate*

The results of the unadjusted analyses of Hu were qualitatively identical to the analyses adjusted for ROWPVT, RPM and participant gender, except that in the unadjusted analyses there was evidence for a main effect of group. The 3-way ANOVA supported main effects of group (*F*(1,125) = 15.61, *p* < .001, ɳ^2^ = .030), emotion (*F*(5,625) =141.47, *p* < .001, ɳ^2^ = .141) and intensity (*F*(7,875) = 472.54, *p* < .001, ɳ^2^ = .427) and interactions between group and emotion (*F*(5,625) = 4.51, *p* < .001, ɳ^2^ = .005), group and intensity (*F*(7,875) = 5.30, *p* < .001, ɳ^2^ = .008), emotion and intensity (*F*(35,4375) = 14.46, *p* < .001, ɳ^2^ = .045), and group, emotion and intensity (*F*(35,4375) = 1.53, *p* = .022, ɳ^2^ = .005).

Follow up 2-way ANOVAs supported a main effect of group for all emotions (*F*(1,125) > 4.73, *p* < .031, ɳ^2^ > .015), except perhaps surprise (*F*s(1,125) = 3.13, *ps* = .079, ɳ^2^ = .010). There was evidence for a main effect of intensity for all emotions (*F*s(7,875) > 54.59, *p*s < .001, ɳ^2^ > .205) and evidence for interactions for anger (*F*(7,875) = 2.58, *p* = .012, ɳ^2^ = .013), disgust (*F*(7,875) = 5.90, *p* < .001, ɳ^2^ = .027), sadness (*F*(7,875) = 3.24, *p* = .002, ɳ^2^ = .016) and surprise (*F*(7,875) = 2.06, *p* = .046, ɳ^2^ = .010), and weak evidence for an interaction for happiness (*F*(7,875) = 1.72, *p* = .10, ɳ^2^ = .008), but not fear (*F*(7,875) = 1.07, *p* = .37, ɳ^2^ = .005).

*Unbiased hit rate analyses excluding participants based on SCQ cut-offs*

As stated in the manuscript, the adjusted analysis with participants excluded based on the SCQ cut-off was broadly similar to adjusted analysis of the full sample. The only difference was that the evidence for interactions between group and intensity in the follow up analyses for separate emotions was somewhat weaker. The 3-way ANCOVA supported main effects of emotion (*F*(5,565) = 129.81, *p* < .001, ɳ^2^ = .148) and intensity (*F*(7,791) = 425.75, *p* < .001, ɳ^2^ = .439) but not group (*F*(1,113) = 0.14, *p* = .71, ɳ^2^ < .001). There was evidence for interactions between group and emotion (*F*(5,565) = 4.29, *p* < .001, ɳ^2^ = .006), group and intensity (*F*(7,791) = 4.30, *p* < .001, ɳ^2^ = .008), emotion and intensity (*F*(35,3955) = 12.66, *p* < .001, ɳ^2^ = .045), and group, emotion and intensity (*F*(35, 3955) = 1.54, *p* = .022, ɳ^2^ = .006).

Follow up 2-way ANCOVAs provided evidence for a main effect of intensity for all emotions (*F*s(7,791) > 52.35, *p*s < .001, ɳ^2^ > .224). There was some evidence for a main effect of group for disgust (*F*(1,113) = 3.57, *p* = .061, ɳ^2^ = .013 but no other emotions (*F*s(1,113) < 0.54, *ps* > .46, ɳ^2^ < .002). There was evidence for interactions between group and intensity for anger (*F*(7,791) = 2.61, *p* = .012, ɳ^2^ = .014), disgust (*F*(7,791) = 5.33, *p* < .001, ɳ^2^ = .026) and sadness (*F*(7,791) = 2.28, *p* = .026, ɳ^2^ = .013), and weak evidence an interaction between group and intensity for happiness (*F*(7,791) = 1.96, *p* = .059, ɳ^2^ = .010) and perhaps surprise (*F*(7,791) = 1.45, *p* = .182, ɳ^2^ = .008) but not fear (*F*(7,791) = 1.17, *p* = .32, ɳ^2^ = .006).

*Unbiased hit rate analysis excluding females*

The analysis with female participants excluded was broadly similar to analysis that included males and females. Again, the only difference was that the evidence for interactions between group and intensity in the follow up analyses for separate emotions was weaker. The 3-way ANCOVA supported main effects of emotion (*F*(5,415) = 81.89, *p* < .001, ɳ^2^ = .133) and intensity (*F*(7,581) = 301.02, *p* < .001, ɳ^2^ = .414) but not group (*F*(1,83) = 1.57, *p* = .21, ɳ^2^ < .004). There was evidence for interactions between group and emotion (*F*(5,415) = 3.81, *p* = .002, ɳ^2^ = .007), group and intensity (*F*(7,581) = 3.53, *p* = .001, ɳ^2^ = .008), emotion and intensity (*F*(35,2905) = 8.45, *p* < .001, ɳ^2^ = .042), and group, emotion and intensity (*F*(35, 2905) = 1.56, *p* = .019, ɳ^2^ = .008).

Follow up 2-way ANCOVAs provided evidence for a main effect of intensity for all emotions (*F*s(7,581) > 33.31, *p*s < .001, ɳ^2^ > .209). There was evidence for a main effect of group for disgust (*F*(1,83) = 9.58, *p* = .003, ɳ^2^ = .047 but no other emotions (*F*s(1,83) < 0.76, *ps* > .38, ɳ^2^ < .004). There was evidence for an interaction between group and intensity for disgust (*F*(7,581) = 5.91, *p* < .001, ɳ^2^ = .039) and some evidence for an interaction between group and intensity for sadness (*F*(7,581) = 1.88, *p* = .070, ɳ^2^ = .015) but little evidence for anger (*F*(7,581) = 1.50, *p* = .165, ɳ^2^ = .011), happiness (*F*(7,581) = 1.41, *p* = .199, ɳ^2^ = .010), surprise (*F*(7,581) = 1.62, *p* = .127, ɳ^2^ = .012) or fear (*F*(7,581) = 0.88, *p* = .320, ɳ^2^ = .007).

*Raw hit rate analyses*

The results of the analysis of raw hit rate were broadly similar to that of unbiased hit rate. The only difference was that the evidence for interactions between group and intensity in the follow up analyses for separate emotions was weaker. There was evidence for main effects of emotion (*F*(5,625) = 92.94, *p* < .001, ɳ^2^ = .162) and intensity (*F*(7,875) = 581.16, *p* < .001, ɳ^2^ = .349) but not group (*F*(1,125) = 1.10, *p* = .30, ɳ^2^ = .001). There was evidence for interactions between group and emotion (*F*(5,625) = 3.81, *p* = .002, ɳ^2^ = .007), group and intensity (*F*(7,875) = 3.44, *p* = .001, ɳ^2^ = .003), emotion and intensity (*F*(35,4375) = 20.38, *p* < .001, ɳ^2^ = .072) and group, emotion and intensity (*F*(35,4375) = 1.41, *p* = .057, ɳ^2^ = .005).

In the follow up analyses by emotion there was evidence for a main effect of intensity for all emotions (*F*s(7,875) > 20.34, *p*s < .001, ɳ^2^ > .083) and evidence of a main effect of group for recognition of disgust (*F*(1,125) = 8.37, *p* = .005, ɳ^2^ = .030) but other emotions (*F*s(1,125) < 0.42, *ps* > .519, ɳ^2^ < .002). There was evidence for interactions between group and intensity for sadness (*F*(7,875) = 2.27, *p* = .027, ɳ^2^ = .010) and surprise (*F*(7,875) = 3.28, *p* = .001 , ɳ^2^ = .015) and weak evidence for the interaction for disgust (*F*(7,875) = 1.72, *p* = .101, ɳ^2^ = .007). However, in contrast to the analysis of unbiased hit rate there was little evidence of an interaction for anger (*F*(7,875) = 1.14, *p* = .333, ɳ^2^ = .006), happiness (*F*(7,875) = 0.67, *p* = .741, ɳ^2^ = .003) or fear (*F*(7,875) = 1.48, *p* = .169, ɳ^2^ = .007).
